# Interpretable machine learning model for predicting postoperative hypoproteinemia in middle-aged and elderly patients following total joint arthroplasty: a multicenter retrospective study

**DOI:** 10.3389/fnut.2026.1775504

**Published:** 2026-06-12

**Authors:** Xiao Chen, Jing Chen, Chang Chen, Haibo Gong, Yuan Luo, Qi Huang, Zhihui Wei, Zhe Wu

**Affiliations:** 1 Department of Orthopedics, The First People's Hospital of Neijiang, Neijiang, Sichuan, China; 2 Department of Pediatrics, The First People's Hospital of Neijiang, Neijiang, Sichuan, China; 3 Department of Rehabilitation, The First People's Hospital of Neijiang, Neijiang, Sichuan, China; 4 Department of Oncology, The First People's Hospital of Neijiang, Neijiang, Sichuan, China; 5 Department of Orthopedics, Yongchuan Hospital of Chongqing Medical University, Chongqing, China; 6 Department of Radiology, The First People's Hospital of Neijiang, Neijiang, Sichuan, China

**Keywords:** hypoproteinemia, machine learning, prediction model, SHapley Additive exPlanations, total joint arthroplasty

## Abstract

**Background:**

Postoperative hypoproteinemia is a prevalent yet frequently overlooked complication following total hip or knee arthroplasty (TJA), adversely impacting wound healing and postoperative recovery in patients. This study aims to identify and evaluate the risk factors associated with hypoproteinemia in middle-aged and elderly patients after TJA, as well as to develop and validate an interpretable machine learning model for predicting high-risk patients promptly.

**Materials and method:**

This study employed a multicenter retrospective research design, gathering clinical data from middle-aged and elderly patients who underwent TJA surgery across two hospitals. Key predictors were identified and selected using minimum absolute shrinkage and selection operator (LASSO) regression. The predictive performance of eight machine learning algorithms was compared, and the SHapley Additive exPlanations (SHAP) analysis was conducted to rank the features and assess the interpretability of the optimal model.

**Results:**

A total of 1,383 participants were evaluated, including 585 patients (mean age: 65 ± 10; 233 men) in the training cohort, 250 patients (mean age: 65 ± 9; 101 men) in the internal validation cohort, and 548 patients (mean age: 69 ± 10; 186 men) in the external validation cohort. Key predictors identified include age, surgery time, anesthesia method, erythrocyte sedimentation rate (ESR), and serum calcium level. Notably, the Light Gradient Boost Machine (LightGBM) model exhibited the highest predictive performance, with AUC values of 1.000, 0.968, and 0.855 in the training, internal validation, and external validation sets, respectively. Consequently, an interpretable LightGBM model incorporating five variables was established. SHAP analysis showed that age, ESR, surgery time, serum calcium level and anesthesia method made significant contributions to the prediction of postoperative hypoproteinemia. Finally, we present the model as an accessible web-based tool for individualized, real-time clinical decision-making (https://hypoproteinemia-prediction-uzhpp3jadq8ryey4jxq3qe.streamlit.app/).

**Conclusion:**

This study successfully developed and validated a machine learning prediction model that exhibit high accuracy and interpretability. This model demonstrates high accuracy and interpretability in predicting the risk of hypoproteinemia following TJA and elucidates intricate nonlinear relationships among various factors. It may serve as a promising tool for early risk screening and proactive prevention, though further prospective validation is needed before routine clinical implementation.

## Introduction

Total joint replacement (TJA), encompassing total hip arthroplasty (THA) and total knee arthroplasty (TKA), represents the primary surgical intervention for end-stage hip and knee joint diseases, including severe osteoarthritis, rheumatoid arthritis, traumatic osteoarthritis, and femoral head necrosis, etc. (1, 2). The principal aim of TJA is to significantly alleviate pain, correct limb deformities, and restore joint function through the implantation of artificial prostheses that replace severely damaged hip and knee joint structures resulting from disease or injury. For middle-aged and elderly patients afflicted by these conditions, TJA serves not only as a technical means to enhance mobility but also as a crucial strategy to fundamentally improve quality of life, facilitate social participation, and reduce the burden of family caregiving (3). In recent decades, the number of TJA performed worldwide has experienced continuous and rapid growth, driven by population aging, advancements in surgical techniques, and improvements in prosthetic materials (4). Projections indicate that by 2040, the number of primary THA and TKA will reach 2.79 million and 2.17 million cases, respectively (5). Notably, the majority of patients undergoing these procedures are middle-aged or elderly individuals, many of whom present with multiple underlying health conditions. This demographic underscores the importance of effective perioperative safety management and the prevention and treatment of complications, as these factors directly influence the success of the surgery and the quality of patient recovery (6). Among the various complications, nutritional metabolic disorders, which may arise from surgical stress, high energy expenditure, and inadequate nutritional intake, are increasingly recognized in clinical practice. Hypoproteinemia, in particular, is a common yet often overlooked postoperative complication that warrants attention (7, 8).

Postoperative hypoproteinemia, characterized by serum albumin levels below 35 g/L (9) (clinical intervention typically necessary below 30 g/L (10)), is not a standalone condition but rather a pronounced indication of negative nitrogen balance and nutrient depletion following major surgical procedures. The etiology of this phenomenon is intricate: surgical trauma induces systemic inflammatory response syndrome, prompting a hypercatabolic state where protein breakdown surpasses synthesis. Concurrently, heightened capillary permeability results in albumin leakage into interstitial spaces. When compounded with intraoperative and postoperative blood loss, hemodilution, and inadequate nutrient uptake, these factors collectively precipitate a steep decline in serum albumin levels. This metabolic anomaly poses a significant, comprehensive threat to patients’ postoperative recovery across multiple systems (11, 12). Functionally, albumin plays a pivotal role in sustaining lymphocyte proliferation and immunoglobulin synthesis; thus, reduced albumin levels impair cellular and humoral immune functions, markedly elevating the susceptibility to deep surgical site infections, periprosthetic infections, pulmonary infections, and urinary tract infections-a dire complication particularly concerning TJA (13, 14). At the tissue healing level, albumin deficiency, the primary substance upholding plasma’s colloid osmotic pressure, can induce interstitial edema, detrimentally impacting the vascularity and regenerative capacity of surgical incisions and deep soft tissues. This can result in compromised incision healing, exudation, delayed recovery, and potential rupture, providing an entry point for pathogenic bacteria (15, 16). Concerning the rehabilitation phase, hypoproteinemia often manifests alongside fatigue, lethargy, and muscle wasting, impeding patients from engaging in early rehabilitation exercises, prolonging immobilization, and heightening the susceptibility to deep vein thrombosis and pulmonary embolism. This sets off a detrimental cycle, culminating in extended hospitalizations and a substantial escalation in healthcare costs. Hypoalbuminemia is linked to a range of adverse postoperative outcomes. Shibata et al. demonstrated that in patients undergoing cardiac surgery, improved postoperative albumin levels (*Δ*-albumin) correlated with reduced mortality (HR = 0.62, 0.52–0.75) (17). Kyun-Ho Shin et al. reported that early postoperative hypoalbuminemia serves as an independent risk factor for acute kidney injury (18) and pneumonia (19) following hip fracture surgery in elderly patients. Additionally, Yu Yibing et al. identified hypoalbuminemia as a significant risk factor for pulmonary infections after hip joint surgery in the elderly, which severely impedes postoperative recovery and may even result in patient mortality (20). Furthermore, Gong et al. (21) found that hypoalbuminemia following THA in elderly patients with femoral neck fractures heightens the risk of postoperative infections and extends hospital stays. This study also confirmed that advanced age, lower BMI, longer operation duration, preoperative blood calcium levels, and preoperative ESR are independent risk factors for postoperative hypoalbuminemia in elderly patients with femoral neck fractures. It is worth noting that currently, the clinical intervention for hypoproteinemia after TJA is mainly “passive correction,” that is, after the patient’s serum albumin decreases, it is treated by supplementing albumin, adjusting the nutritional support plan, etc. While human serum albumin is beneficial in shock scenarios like blood loss, trauma, and burns, its efficacy may be limited in elderly patients with chronic conditions such as chronic renal failure. Hence, investigating the etiology and early warning indicators of hypoproteinemia post-joint replacement in middle-aged and elderly individuals is essential for prompt intervention and the prevention of adverse outcomes linked to these complications.

Currently, there is a lack of systematic approaches for identifying risk factors associated with hypoproteinemia following TJA, hindering clinicians in accurately screening high-risk patients and implementing targeted preventive measures. In recent years, machine learning (ML) algorithms such as Random Forest (RF), Extreme Gradient Boosting (XGBoost), and Support Vector Machines (SVMs) have gained significant attention for developing clinical prediction models. Unlike traditional linear models, ML models can effectively manage complex nonlinear relationships and high-dimensional data, thereby improving predictive performance (22, 23). Nonetheless, ML technology presents some limitations. A notable drawback is the complexity and lack of interpretability of these models, which often leads to their characterization as “black boxes (24).” To tackle this issue, this study employs the SHapley Additive exPlanations (SHAP) method to interpret the machine learning model and visualize the predictive contributions of each variable (25). Consequently, this study aims to develop and validate a machine learning model that integrates relevant preoperative and intraoperative clinical indicators to predict the risk of hypoproteinemia after TJA in middle-aged and elderly patients. Furthermore, the introduction of visualization tools has improved the transparency of the decision-making process in ML model, thereby enhancing clinicians’ trust in the predictive outcomes and facilitating the formulation of individualized treatment strategies.

## Methods

### Study design and participants

We retrospectively analyzed data from 835 middle-aged and elderly patients who received TJA at our hospital’s joint surgery department between January 2020 and August 2025. The participants were then randomly allocated into training set consisting of 585 individuals and internal validation set consisting of 250 individuals, maintaining a ratio of 7:3. Model development and internal test were performed. Additionally, 548 patients who underwent TJA at another hospital’s joint surgery department from January 2022 to August 2025 were included for external validation. This multi-center retrospective study adhered to the 1964 Helsinki Declaration and obtained ethical approval from the respective ethics committees of the two hospitals. Informed consent requirement for each participant was waived by the ethics committee due to the retrospective nature of the study. This multi-center retrospective study was conducted using electronic medical records from two hospitals (January 2020 to August 2025). Data extraction was performed independently by two researchers, with discrepancies resolved by a third senior author. To ensure data quality, variables with >5% missingness were excluded from analysis, and participants with any missing values in the remaining variables were removed (complete case analysis). The study design and reporting followed the TRIPOD checklist.

Sample size justification: The minimum required sample size was estimated based on the “10 events per predictor variable (EPV)” rule. A total of 37 candidate variables were initially considered for model development, requiring at least 370 hypoproteinemia events for adequate statistical power. In the primary hospital cohort (*n* = 835), 452 patients (54.13%) developed postoperative hypoproteinemia, which exceeded the required threshold. Therefore, the sample size was sufficient for reliable model development and internal validation.

**Figure 1:**
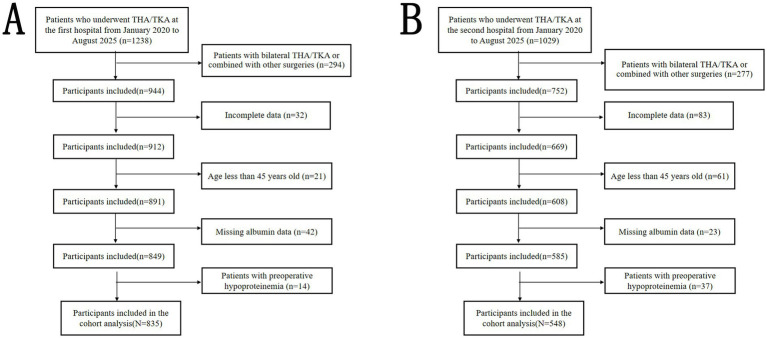
The flowchart of participant selection process.

The study’s inclusion criteria are: (1) Age ≥45 years; (2) Elective surgery necessitated by conditions such as femoral head necrosis, osteoarthritis, etc.; (3) Complete medical records; and (4) Absence of underlying diseases related to the liver or kidneys. The exclusion criteria are: (1) Surgical causes including hip fracture, rheumatoid arthritis, tumors, or pathological fractures, etc.; (2) Preoperative hypoproteinemia (serum albumin level < 35 g/L); (3) Moderate to severe preoperative malnutrition; (4) Simultaneous bilateral joint replacement surgery or combined surgeries (e.g., fracture internal fixation, internal fixation removal); (5) Receipt of preoperative human albumin injections; (6) Other chronic wasting diseases; and (7) Incomplete or missing clinical data. The research process is illustrated in Figure 1.

### Data collection

Candidate variables were selected based on a review of published literature on risk factors for postoperative hypoproteinemia in patients undergoing major orthopedic surgery, as well as clinical judgment. The following variables were extracted from the electronic medical records of all participants, encompassing various demographic and clinical parameters, including:

*Demographic and clinical characteristics:* age, gender, height, weight, body mass index (BMI), hypertension, diabetes, cardiovascular disease, hyperlipidemia, hyperuricemia, American Society of Anesthesiologists score (ASA), anesthesia method, surgical method, and operation time.

*Preoperative laboratory indicators:* white blood cell count (WBC), neutrophil count (NC), lymphocyte count (LC), monocyte count (MC), red blood cell count (RBC), hemoglobin (HGB), preoperative platelet count (PLT), hematocrit (HCT), and mean red blood cell volume (MCV). Additional parameters consist of mean corpuscular hemoglobin (MCH), mean corpuscular hemoglobin concentration (MCHC), prothrombin time (PT), activated partial thrombin time (APTT), international normalized ratio (INR), thrombin time (TT), fibrinogen (FIB), aspartate aminotransferase (AST), alanine aminotransferase (ALT), blood calcium, C-reactive protein (CRP), erythrocyte sedimentation rate (ESR), and albumin level (Alb).

*Inflammation and nutrition-related indicators:* neutrophil-to-lymphocyte ratio (NLR), platelet-to-lymphocyte ratio (PLR), lymphocyte-to-monocyte ratio (LMR), systemic immune-inflammation index (SII), systemic inflammatory response index (SIRI), pan-immune inflammation value (PIV), advanced lung cancer inflammation index (ALI), NAR, CAR, prognostic nutritional index (PNI), and high-sensitive modified Glasgow prognostic score (HS-mGPS). As previously described (26–30), the following formulas were utilized:

- NLR = neutrophil/lymphocyte.
- PLR = platelet/lymphocyte.
- LMR = lymphocyte/monocyte.
- SII = (neutrophil * platelet)/lymphocyte.
- SIRI = (neutrophil * monocyte)/lymphocyte.
- PIV = (neutrophil * platelet * monocyte)/lymphocyte.
- ALI = (BMI * ALB)/NLR.
- NAR = neutrophil/Alb.
- CAR = CRP/Alb.
- PNI = ALB (g/L) + 5 × LC (×10^9^/L).

HS-mGPS: Calculated from Alb and CRP levels, with scores assigned as 0 points for CRP ≤ 3 mg/L, 1 point for CRP > 3 mg/L and ALB ≥ 35 g/L, and 2 points for CRP > 3 mg/L and ALB < 35 g/L. HS-mGPS ≥ 1 point indicates malnutrition.

### Feature selection

The candidate variables considered for model development were selected based on a comprehensive literature review and clinical expertise. Specifically, we searched PubMed and Web of Science for studies on hypoproteinemia following major orthopedic surgery and identified previously reported risk factors, including demographic characteristics (age, sex, BMI), comorbidities (hypertension, diabetes, cardiovascular disease), surgical factors (anesthesia method, operation time), and preoperative laboratory indicators (inflammatory markers, nutritional parameters, blood cell counts, coagulation function, and serum calcium). A total of 37 variables were initially included based on their availability in the electronic medical records of both participating hospitals. All candidate variables were extracted from routine preoperative assessments documented in the electronic medical records.

A prediction model for postoperative hypoalbuminemia risk was developed using various methods. Firstly, the 835 participants in the first hospital were randomly divided into training set (585 participants) and internal validation set (250 participants) at a 7:3 ratio. The training set was utilized for model development, while the internal validation set was used for internal validation, and the datasets from the second hospital (548 participants) for external validation. The least absolute contraction and selection operator (LASSO) logistic regression analysis was employed to identify potential predictors of postoperative hypoalbuminemia risk. LASSO regression aids in addressing collinearity among covariates and selecting the most predictive risk factors by utilizing L1 regularization, tuning parameters (*λ* values), and cross-validation. Cross-validation involves shrinkage through penalizing the absolute value of the coefficient size (L1 penalty), effectively reducing the coefficients of irrelevant features to zero. This approach facilitates feature selection, model simplification, and the identification of optimal variables. An optimal LASSO model was then developed based on the selected variables. A multivariate logistic regression model was constructed for the non-zero coefficient variables identified in the LASSO regression model. Variables that remained significantly correlated with postoperative hypoalbuminemia risk in the multivariate model were retained.

Rationale for LASSO regression: LASSO regression was chosen for feature selection because it applies L1 regularization, which shrinks irrelevant feature coefficients to zero, effectively addressing multicollinearity among the 37 candidate variables. This approach balances model simplicity and predictive power, making it particularly suitable when the number of candidate variables is relatively large compared to the sample size.

### Model development, comparisons, and evaluation

Choice of machine learning algorithms: The eight selected algorithms represent diverse modeling paradigms, each with distinct strengths for clinical prediction tasks. Logistic Regression (LR) was included as a parametric linear baseline, providing interpretable coefficients and serving as a reference for evaluating the added value of more complex models. K-Nearest Neighbors (KNN) is a non-parametric instance-based learner that makes predictions based on similarity to nearest neighbors in the feature space, allowing it to capture local patterns without assuming a specific functional form. Decision Tree (DT) offers a simple rule-based structure that is highly transparent and easy to visualize, though it is prone to overfitting. To overcome the limitations of single trees, we included four ensemble methods: Random Forest (RF) uses bagging to reduce variance and improve stability; Extreme Gradient Boosting (XGBoost), Light Gradient Boost Machine (LightGBM), and Categorical Boosting (CatBoost) represent gradient boosting frameworks that sequentially correct prediction errors, each with distinct optimization strategies—XGBoost with regularization and sparse data handling, LightGBM with leaf-wise growth for faster training on larger datasets, and CatBoost with native support for categorical features and reduced overfitting. Finally, Support Vector Machine (SVM) is a kernel-based method that maps features into higher-dimensional spaces to capture complex nonlinear boundaries. These eight models (LR, KNN, DT, RF, XGBoost, LightGBM, CatBoost, and SVM) were constructed to predict postoperative hypoproteinemia. By comparing algorithms with such diverse underlying mechanisms, we aimed to identify the model that best captures the complex, nonlinear relationships between the selected predictors and the risk of postoperative hypoproteinemia, while also assessing trade-offs between predictive performance, interpretability, and computational efficiency.

Rationale for 5-fold cross-validation: Five-fold cross-validation was chosen as a trade-off between bias and variance. Compared with leave-one-out cross-validation, 5-fold cross-validation reduces computational cost while maintaining robust performance estimation. Compared with a single train-test split, it mitigates overfitting by averaging results across multiple data partitions. This approach is widely recommended for moderate-sized datasets (*n* = 585) in clinical prediction model studies. Therefore, to minimize overfitting and enhance model robustness before external validation, 5-fold cross-validation was performed on the derivation cohort.

To select the best predictive model, we empirically defined an exploratory composite metric, referred to as *AF_score_*, which integrates the Area Under the Receiver Operating Characteristic Curve (AUROC) and the F1 score to comprehensively assess the model’s discriminative and calibration capabilities. To avoid over-optimistic estimation caused by the training data, the model evaluation did not use the performance indicators of the training set, but relied solely on internal cross-validation and external validation indicators to reliably assess the model’s generalization ability. The mathematical formula of *AF_score_* is shown in Equation 1.


$$AF_{\text{score}=}\;\alpha *\;\bar{\text{AUROC}}+\alpha *\;\bar{F1}$$ (1)

α=0.5 (2)

Where $\bar{\text{AUROC}}$ and $\bar{F1}$ represents the arithmetic mean of the AUROC value and F1 score from the internal cross-validation and external validation sets, respectively. By setting α = 0.5, equal emphasis is placed on discrimination and calibration. We acknowledge that this composite metric is exploratory and requires independent methodological validation. Nevertheless, it served as a key integrated measure for model comparison in this study, alongside other established metrics including AUROC, area under the Precision-Recall curve (AUPRC), calibration curves, and decision curve analysis (DCA).

The discrimination ability of the final model was assessed using the receiver operating characteristic (ROC) curve and Precision-Recall (PR) curve, while calibration was evaluated with the calibration curve. In addition, DCA was used to evaluate the net benefit of the model at different thresholds.

### Model explanation

ML models excel in predictive tasks, yet their opacity and lack of interpretability in decision-making processes can instill skepticism among clinicians, hindering widespread adoption in clinical settings. To enhance model reliability and acceptance, we employ the SHAP method rooted in game theory to visually scrutinize and elucidate the optimal machine learning model. SHAP was selected for model interpretability because it provides a unified framework based on game theory, ensuring desirable properties such as consistency and local accuracy. Unlike traditional feature importance measures (e.g., permutation importance), SHAP quantifies both the direction (positive/negative) and magnitude of each feature’s contribution to individual predictions. Furthermore, SHAP is particularly well-suited for tree-based models like LightGBM, offering computational efficiency and global interpretability through summary plots, dependence plots, and force plots. Through assessing the impact of each feature on individual predictions, SHAP facilitates quantitative evaluation of overall feature importance and offers intuitive interpretations for individual instances. It is one of the latest advanced methods for interpreting tree models. Within this framework, the model’s raw output value *f*(*x*) for a sample (representing the log-odds in a binary classification model) can be converted into a probability value (*p* = 1/(1 + e^ (−*f*(*x*)))) through a sigmoid function. This transformation allows for comparison with the diagnostic threshold for binary classification decisions: a larger *f*(*x*) value leans towards classifying the sample as “postoperative hypoproteinemia,” while a smaller value leans towards “non-postoperative hypoproteinemia.” Through SHAP, we enhance model transparency and elucidate each feature’s distinct contribution to individual predictions, thereby improving the model’s interpretability and clinical utility.

### Statistical analysis

Statistical analysis of the data was conducted using R (version 4.2.2) and Python (version 3.10.2). Continuous variables were presented as mean ± standard deviation (SD) and group differences were assessed using t-tests. Categorical variables were depicted as frequencies and percentages and compared utilizing chi-square tests. All analyses were two-tailed, and statistical significance was set at *p* < 0.05. All statistical analyses were performed in accordance with the study objectives. Detailed justifications for feature selection, model development, and model interpretation are provided in the preceding sections.

## Results

### Baseline characteristics of the participants

After a stepwise screening process, the study included 1,383 participants who underwent TJA and met the specified inclusion and exclusion criteria in those two hospitals (835 cases in the first hospital and 548 in the second hospital). For the first datasets, postoperative hypoproteinemia occurred in 452 participants (452/835), with an incidence rate of 54.13%. The participants were then randomly allocated into training set consisting of 585 individuals and internal validation set consisting of 250 individuals, maintaining a ratio of 7:3. The mean age of the participants in the training set was 65 ± 10 years, with 325 hypoproteinemia (55.56%). In comparison, the mean age of the internal validation set was 65 ± 9 years, with 127 hypoproteinemia (50.80%). In this study, we analyzed baseline demographic and clinical characteristics of different cohorts of participants. No significant differences were observed in any parameter between the training and internal validation cohorts (*p* > 0.05), indicating that the baseline characteristics were comparable between the two groups (Table 1). The baseline characteristics of the external validation datasets is shown in Supplementary Table 1.

**Table 1:** Patient demographics and baseline characteristics in training and internal cohorts.

Characteristic	Cohort		*p*-value
	Training cohort, *N* = 585	Internal test cohort, *N* = 250	
Gender, *n* (%)			0.877
Male	233 (39.8%)	101 (40.4%)	
Female	352 (60.2%)	149 (59.6%)	
Age (years), Mean ± SD	65 ± 10	65 ± 9	0.799
BMI (Kg/m^2^), Mean ± SD	24.7 ± 4.2	25.3 ± 8.9	0.345
Hypertension, *n* (%)			0.915
Yes	178 (30.4%)	77 (30.8%)	
No	407 (69.6%)	173 (69.2%)	
Diabetes, *n* (%)			0.992
Yes	68 (11.6%)	29 (11.6%)	
No	517 (88.4%)	221 (88.4%)	
Cardiovascular disease, *n* (%)			0.185
Yes	34 (5.8%)	9 (3.6%)	
No	551 (94.2%)	241 (96.4%)	
Hyperlipidaemia, *n* (%)			0.829
Yes	35 (6.0%)	14 (5.6%)	
No	550 (94.0%)	236 (94.4%)	
Hyperuricemia, *n* (%)			0.795
Yes	161 (27.5%)	71 (28.4%)	
No	424 (72.5%)	179 (71.6%)	
ASA, *n* (%)			0.503
≥III	117 (20.0%)	45 (18.0%)	
<III	468 (80.0%)	205 (82.0%)	
General anesthesia, *n* (%)			0.440
Yes	36 (6.2%)	19 (7.6%)	
No	549 (93.8%)	231 (92.4%)	
Surgical method, *n* (%)			0.521
THA	276 (47.2%)	124 (49.6%)	
TKA	309 (52.8%)	126 (50.4%)	
Surgery time (minutes), Mean ± SD	98 ± 30	95 ± 33	0.211
RBC, Mean ± SD	4.26 ± 0.52	4.26 ± 0.53	0.869
HGB, Mean ± SD	128 ± 16	128 ± 16	0.450
MCV, Mean ± SD	92 ± 7	92 ± 8	0.856
MCH, Mean ± SD	30.10 ± 2.66	31.44 ± 18.51	0.255
MCHC, Mean ± SD	326 ± 17	324 ± 31	0.595
NLR, Mean ± SD	2.80 ± 1.52	2.76 ± 1.54	0.721
PLR, Mean ± SD	131 ± 62	127 ± 60	0.410
LMR, Mean ± SD	4.06 ± 11.77	3.63 ± 1.41	0.388
SII, Mean ± SD	537 ± 353	510 ± 355	0.300
SIRI, Mean ± SD	1.36 ± 0.91	1.55 ± 3.00	0.333
PIV, Mean ± SD	268 ± 222	315 ± 976	0.455
ALI, Mean ± SD	493 ± 624	481 ± 238	0.685
PT, Mean ± SD	10.70 ± 0.84	10.70 ± 1.17	0.925
INR, Mean ± SD	0.95 ± 0.09	1.13 ± 1.88	0.139
APTT, Mean ± SD	25.3 ± 13.1	24.4 ± 3.6	0.123
TT, Mean ± SD	17.61 ± 3.23	17.58 ± 1.59	0.837
FIB, Mean ± SD	3.28 ± 0.95	3.25 ± 1.23	0.810
AST/ALT, Mean ± SD	1.16 ± 0.45	1.23 ± 0.52	0.070
Calcium, Mean ± SD	2.37 ± 0.15	2.37 ± 0.15	0.922
CRP, Mean ± SD	6 ± 10	6 ± 8	0.688
ESR, Mean ± SD	21 ± 17	20 ± 18	0.763
PNI, Mean ± SD	51.7 ± 26.2	50.2 ± 4.9	0.175
HS-mGPS, *n* (%)			0.471
0	245 (41.9%)	98 (39.2%)	
1	340 (58.1%)	152 (60.8%)	
NAR, Mean ± SD	0.10 ± 0.03	0.09 ± 0.03	0.383
CAR, Mean ± SD	0.15 ± 0.24	0.15 ± 0.20	0.682

BMI, body mass index; THA, total hip arthroplasty; TKA, total knee arthroplasty; ASA, American Society of Anesthesiologists score; RBC, red blood cell; HGB, hemoglobin; MCV, mean corpuscular volume; MCH, mean corpuscular hemoglobin; MCHC, mean corpuscular hemoglobin concentration; NLR, neutrophil to lymphocyte ratio; PLR, platelet to lymphocyte ratio; LMR, lymphocyte to monocyte ratio; SII, systemic immune-inflammatory index; SIRI, systemic inflammatory response index; PIV, pan-immune-inflammation value; ALI, advanced lung cancer inflammation index; PT, prothrombin time; APTT, activated partial thrombin time; INR, international normalized ratio; TT, thrombin time; FIB, fibrinogen; CRP, C-reactive protein; ESR, erythrocyte sedimentation rate; PNI, Prognostic Nutritional Index; HS-mGPS, High-sensitive Modified Glasgow Prognostic Score; NAR, neutrophil to albumin ratio; CAR, CRP to albumin ratio.

In the training set, either the Wilcoxon test or the chi-square test was utilized to compare the indicators of hypoproteinemia group and non-hypoproteinemia group. The univariate analysis results indicated significant differences among the groups include gender (*p* < 0.001), age (*p* < 0.001), hypertension (*p* < 0.001), cardiovascular disease (*p* < 0.001), ASA (*p* < 0.001), anesthesia method (*p* = 0.004), surgical method (*p* < 0.001), surgery time (*p* < 0.001), RBC (*p* < 0.001), HGB (*p* < 0.001), MCH (*p* = 0.025), PT (*p* = 0.036), FIB (*p* < 0.001), calcium (*p* = 0.013), ESR (*p* < 0.001), and HS-mGPS (*p* = 0.007) (Table 2).

**Table 2:** Results of univariate analysis for training, internal validation cohorts.

Characteristics	Training cohort			Internal test cohort		
	No (*n* = 260)	Yes (*n* = 325)	*p*-value	No (*n* = 123)	Yes (*n* = 127)	*p*-value
Gender, *n* (%)			<0.001			<0.001
Male	129 (48.7%)	104 (32.5%)		62 (52.5%)	39 (29.5%)	
Female	136 (51.3%)	216 (67.5%)		56 (47.5%)	93 (70.5%)	
Age (years), Mean ± SD	59 ± 8	70 ± 8	<0.001	60 ± 8	69 ± 8	<0.001
BMI (Kg/m^2^), Mean ± SD	24.8 ± 4.2	24.7 ± 4.1	0.804	25.0 ± 3.9	25.5 ± 11.6	0.604
Hypertension, *n* (%)			<0.001			0.082
Yes	60 (22.6%)	118 (36.9%)		30 (25.4%)	47 (35.6%)	
No	205 (77.4%)	202 (63.1%)		88 (74.6%)	85 (64.4%)	
Diabetes, *n* (%)			0.324			0.785
Yes	27 (10.2%)	41 (12.8%)		13 (11.0%)	16 (12.1%)	
No	238 (89.8%)	279 (87.2%)		105 (89.0%)	116 (87.9%)	
Cardiovascular disease, *n* (%)			<0.001			0.506
Yes	6 (2.3%)	28 (8.8%)		3 (2.5%)	6 (4.5%)	
No	259 (97.7%)	292 (91.3%)		115 (97.5%)	126 (95.5%)	
Hyperlipidaemia, *n* (%)			0.516			0.738
Yes	14 (5.3%)	21 (6.6%)		6 (5.1%)	8 (6.1%)	
No	251 (94.7%)	299 (93.4%)		112 (94.9%)	124 (93.9%)	
Hyperuricemia, *n* (%)			0.586			0.671
Yes	70 (26.4%)	91 (28.4%)		32 (27.1%)	39 (29.5%)	
No	195 (73.6%)	229 (71.6%)		86 (72.9%)	93 (70.5%)	
ASA, *n* (%)			<0.001			<0.001
≥III	25 (9.4%)	92 (28.8%)		10 (8.5%)	35 (26.5%)	
<III	240 (90.6%)	228 (71.3%)		108 (91.5%)	97 (73.5%)	
General anesthesia, *n* (%)			0.004			0.988
Yes	8 (3.0%)	28 (8.8%)		9 (7.6%)	10 (7.6%)	
No	257 (97.0%)	292 (91.3%)		109 (92.4%)	122 (92.4%)	
Surgical method, *n* (%)			<0.001			0.008
THA	155 (58.5%)	121 (37.8%)		69 (58.5%)	55 (41.7%)	
TKA	110 (41.5%)	199 (62.2%)		49 (41.5%)	77 (58.3%)	
Surgery time (minutes), Mean ± SD	92 ± 27	104 ± 32	<0.001	94 ± 34	97 ± 33	0.446
RBC, Mean ± SD	4.36 ± 0.55	4.17 ± 0.48	<0.001	4.35 ± 0.51	4.18 ± 0.54	0.011
HGB, Mean ± SD	132 ± 17	124 ± 14	<0.001	132 ± 16	125 ± 15	<0.001
MCV, Mean ± SD	92 ± 7	92 ± 7	0.335	93 ± 7	92 ± 8	0.321
MCH, Mean ± SD	30.37 ± 2.70	29.87 ± 2.61	0.025	30.53 ± 2.97	32.26 ± 25.34	0.438
MCHC, Mean ± SD	327 ± 21	325 ± 12	0.244	329 ± 12	321 ± 41	0.034
NLR, Mean ± SD	2.71 ± 1.47	2.88 ± 1.56	0.189	2.70 ± 1.51	2.82 ± 1.57	0.539
PLR, Mean ± SD	129 ± 63	132 ± 61	0.549	119 ± 47	134 ± 69	0.038
LMR, Mean ± SD	3.58 ± 1.27	4.45 ± 15.87	0.328	3.61 ± 1.38	3.65 ± 1.44	0.845
SII, Mean ± SD	532 ± 380	542 ± 329	0.736	463 ± 285	551 ± 405	0.045
SIRI, Mean ± SD	1.36 ± 0.99	1.36 ± 0.83	0.959	1.32 ± 1.08	1.74 ± 3.99	0.245
PIV, Mean ± SD	272 ± 248	266 ± 198	0.749	232 ± 205	389 ± 1,328	0.181
ALI, Mean ± SD	492 ± 251	493 ± 813	0.976	482 ± 237	479 ± 240	0.906
PT, Mean ± SD	10.62 ± 0.86	10.77 ± 0.82	0.036	10.71 ± 0.80	10.68 ± 1.43	0.845
INR, Mean ± SD	0.94 ± 0.10	0.96 ± 0.08	0.074	0.95 ± 0.08	1.28 ± 2.58	0.148
APTT, Mean ± SD	24.8 ± 4.0	25.8 ± 17.3	0.315	24.8 ± 3.4	24.1 ± 3.7	0.121
TT, Mean ± SD	17.87 ± 4.51	17.39 ± 1.45	0.097	17.72 ± 1.39	17.45 ± 1.75	0.182
FIB, Mean ± SD	3.09 ± 0.92	3.43 ± 0.95	<0.001	2.95 ± 0.69	3.53 ± 1.51	<0.001
AST/ALT, Mean ± SD	1.13 ± 0.44	1.18 ± 0.45	0.214	1.14 ± 0.41	1.30 ± 0.60	0.011
Calcium, Mean ± SD	2.39 ± 0.15	2.36 ± 0.14	0.013	2.41 ± 0.16	2.34 ± 0.14	<0.001
CRP, Mean ± SD	6 ± 10	7 ± 10	0.184	5 ± 9	7 ± 8	0.241
ESR, Mean ± SD	15 ± 13	26 ± 18	<0.001	13 ± 11	26 ± 20	<0.001
PNI, Mean ± SD	50.8 ± 4.7	52.4 ± 35.1	0.429	50.0 ± 4.8	50.3 ± 5.0	0.652
HS-mGPS, *n* (%)			0.007			0.136
0	127 (47.9%)	118 (36.9%)		52 (44.1%)	46 (34.8%)	
1	138 (52.1%)	202 (63.1%)		66 (55.9%)	86 (65.2%)	
NAR, Mean ± SD	0.09 ± 0.03	0.10 ± 0.03	0.386	0.09 ± 0.03	0.09 ± 0.03	0.736
CAR, Mean ± SD	0.14 ± 0.24	0.17 ± 0.25	0.135	0.13 ± 0.21	0.16 ± 0.20	0.240

BMI, body mass index; THA, total hip arthroplasty; TKA, total knee arthroplasty; ASA, American Society of Anesthesiologists score; RBC, red blood cell; HGB, hemoglobin; MCV, mean corpuscular volume; MCH, mean corpuscular hemoglobin; MCHC, mean corpuscular hemoglobin concentration; NLR, neutrophil to lymphocyte ratio; PLR, platelet to lymphocyte ratio; LMR, lymphocyte to monocyte ratio; SII, systemic immune-inflammatory index; SIRI, systemic inflammatory response index; PIV, pan-immune-inflammation value; ALI, advanced lung cancer inflammation index; PT, prothrombin time; APTT, activated partial thrombin time; INR, international normalized ratio; TT, thrombin time; FIB, fibrinogen; CRP, C-reactive protein; ESR, erythrocyte sedimentation rate; PNI, Prognostic Nutritional Index; HS-mGPS, High-sensitive Modified Glasgow Prognostic Score; NAR, neutrophil to albumin ratio; CAR, CRP to albumin ratio.

### Selection of main predictors of postoperative hypoalbuminemia

**Figure 2:**
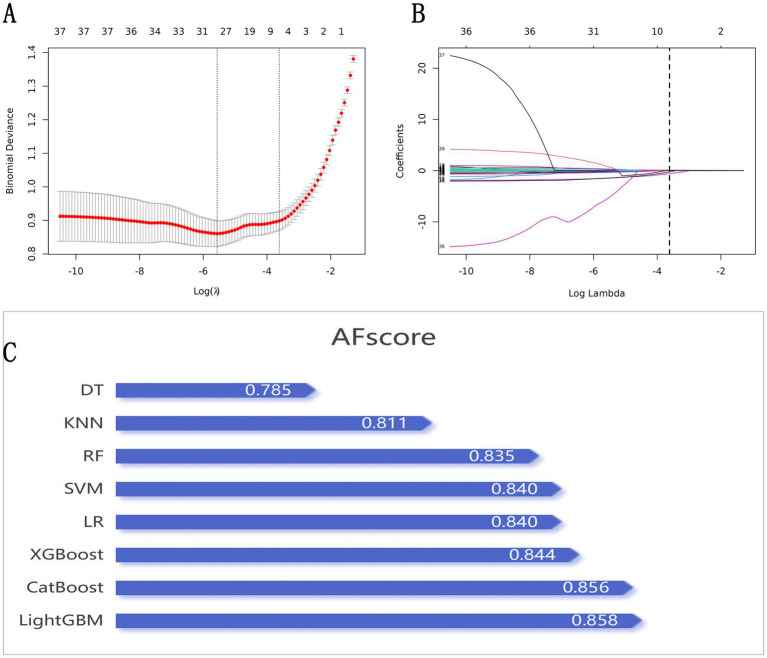
Lasso regression cross-validation plot **(A)** and lasso regression coefficient path plot **(B)**. **(C)** The AF_score_ across different ML models.

LASSO regression analysis was utilized to identify significant factors associated with postoperative hypoalbuminemia. 5 variables were selected from the initial 37 characteristic variables in the training cohort. These variables included age, surgery time, anesthesia method, calcium and ESR, chosen based on their significance in the LASSO model. The variable selection process and coefficients can be observed in Figures 2A,B and Supplementary Table 2, respectively.

### Model development and comparative performance

Based on the selected 5 features, 8 ML models were developed to predict postoperative hypoalbuminemia after TJA. Table 3, Supplementary Figures 1, 2 summarized the performance of these models (LR, KNN, DT, RF, XGBoost, LightGBM, SVM, and CatBoost) based on various evaluation metrics, including AUROC, AUPRC, precision, recall and F1 score. Figure 2C presents a comparison of the *AF_score_* across different ML models. The lightGBM model achieved the highest *AF_score_* (0.858), outperforming all other algorithms. Although some alternative models (e.g., LR and SVM) showed slightly higher external AUROC or F1 scores, the LightGBM model demonstrated superior performance in the internal validation cohort (AUROC: 0.968 vs. 0.906 for LR and 0.907 for SVM; F1: 0.902 vs. 0.820 for LR and 0.825 for SVM) while maintaining acceptable external validity (AUROC: 0.855; F1: 0.706). The integrated *AF_score_*, which balances discrimination (AUROC) and calibration (F1) across both validation sets, therefore, identified LightGBM as the optimal model. Consequently, it was selected as the final model for further evaluation.

**Table 3:** Performance comparison of eight machine learning (ML) models for training, internal valid, and external valid cohorts.

Cohorts	Model	AUC	Accuracy	Precision	Recall	F1 score
Training cohort	RF	0.998	0.973	0.978	0.972	0.975
	XGBoost	1.000	1.000	1.000	1.000	1.000
	LightGBM	1.000	1.000	1.000	1.000	1.000
	CatBoost	0.997	0.966	0.974	0.962	0.968
	LR	0.869	0.765	0.775	0.797	0.786
	DT	1.000	1.000	1.000	1.000	1.000
	SVM	0.869	0.769	0.775	0.807	0.791
	KNN	0.941	0.853	0.886	0.835	0.860
Internal valid cohort	RF	0.969	0.892	0.910	0.890	0.900
	XGBoost	0.960	0.880	0.873	0.912	0.892
	LightGBM	0.968	0.892	0.892	0.912	0.902
	CatBoost	0.973	0.884	0.896	0.890	0.893
	LR	0.906	0.797	0.789	0.853	0.820
	DT	0.862	0.865	0.864	0.890	0.877
	SVM	0.907	0.801	0.787	0.868	0.825
	KNN	0.910	0.821	0.847	0.816	0.831
External valid cohort	RF	0.770	0.715	0.725	0.715	0.701
	XGBoost	0.823	0.714	0.720	0.714	0.701
	LightGBM	0.855	0.719	0.729	0.719	0.706
	CatBoost	0.852	0.719	0.728	0.719	0.706
	LR	0.864	0.785	0.827	0.785	0.769
	DT	0.693	0.723	0.737	0.723	0.707
	SVM	0.875	0.766	0.798	0.766	0.751
	KNN	0.805	0.710	0.717	0.710	0.697

LR, Logistic Regression; KNN, K-Nearest Neighbors; DT, Decision Tree; RF, Random Forest; XGBoost, Extreme Gradient Boosting; CatBoost, Categorical Boosting; LightGBM, Light Gradient Boost Machine; SVM, Support Vector Machine.

### Model evaluation and subgroup performance analysis

**Figure 3:**
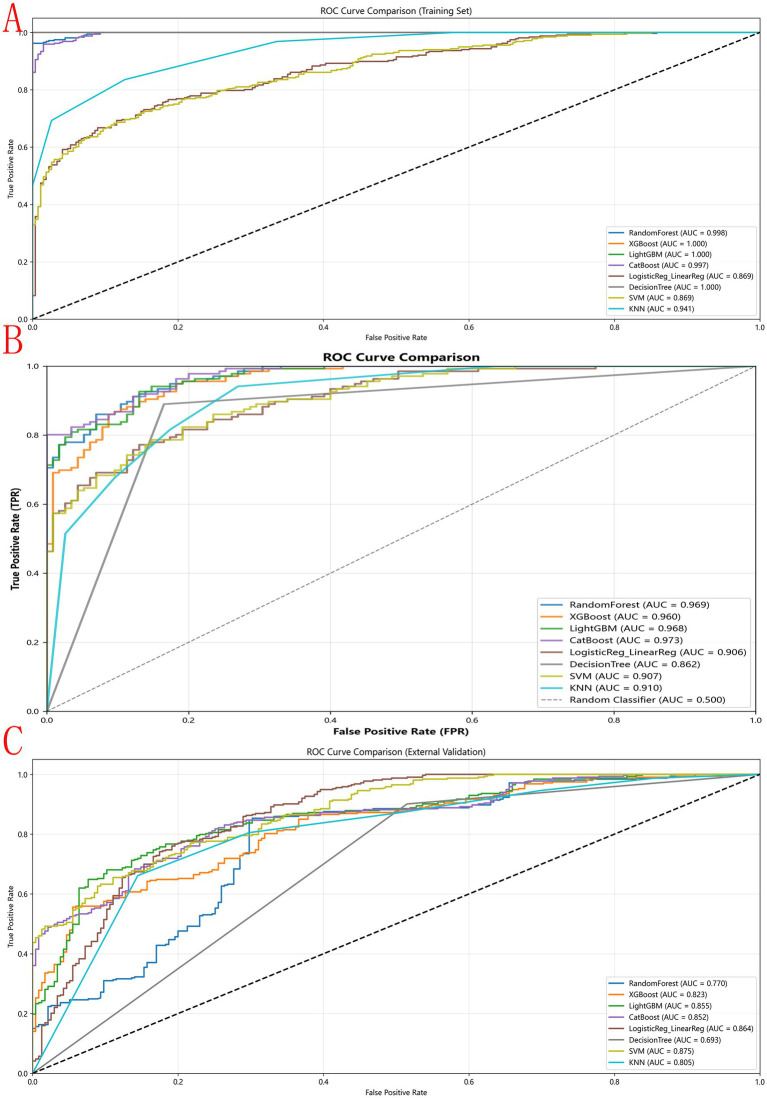
ROC curve of 8 different machine learning models (**A**: training cohort; **B**: internal validation cohort; **C**: external validation cohort).

We comprehensively evaluated the final LightGBM model in terms of both discrimination and calibration. The AUROC was 1.000 in the training cohort (Figure 3A), 0.968 in the internal cross-validation (Figure 3B), and 0.855 in the external validation cohort (Figure 3C). Consistent with AUPRC remain high, with values of 1.000, 0.974, and 0.891, respectively (Supplementary Figure 1).

**Figure 4:**
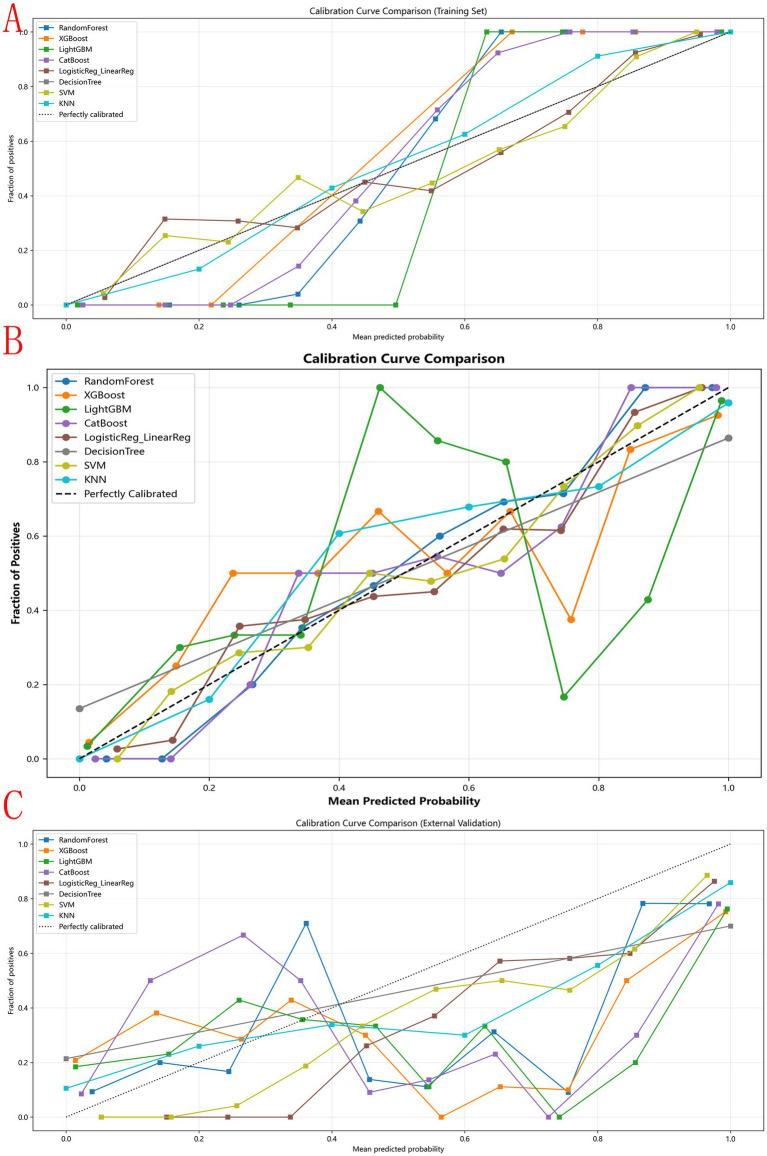
Calibration curves (**A**: training cohort; **B**: internal validation cohort; **C**: external validation cohort) of 8 different machine learning models.

**Figure 5:**
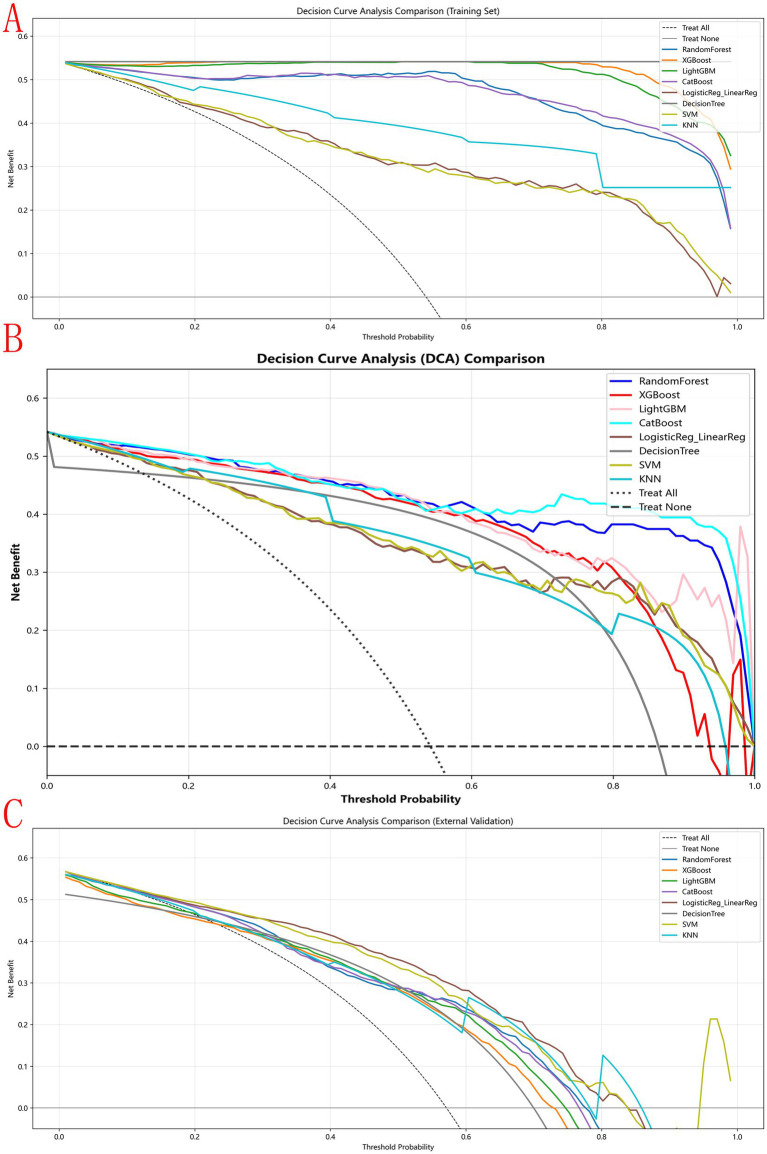
Decision curve analysis (**A**: training cohort; **B**: internal validation cohort; **C**: external validation cohort) of 8 different machine learning models.

Calibration performance is shown in Figure 4, the predicted probabilities demonstrated overall good agreement with the observed outcomes. Slight underestimation was noted at higher predicted risk levels in the external validation cohort. Clinical utility was evaluated using DCA (Figure 5). Across a wide range of threshold probabilities, the model consistently provided a greater net benefit than both the treat-all and treat-none strategies.

### Model interpretability

**Figure 6:**
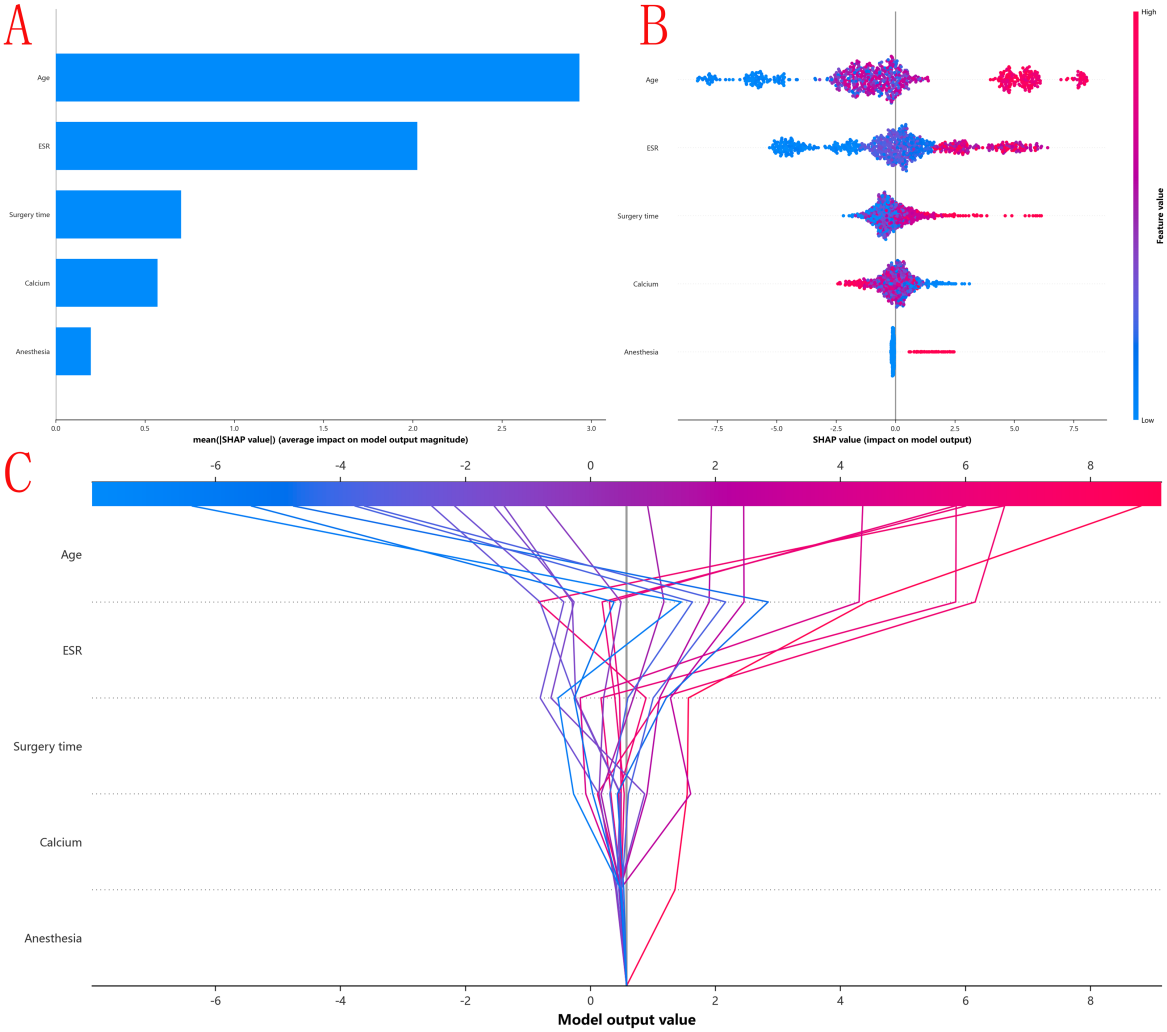
Global model explanation by the SHAP method. **(A)** Bar plot showing the mean absolute SHAP values for each feature, including the overall importance of features in predicting postoperative hypoproteinemia in TJA patients. **(B)** Violin plot illustrating the distribution of SHAP values for each feature. The color gradient represents the feature values (yellow for high values and purple for low values). **(C)** The decision plot.

This study utilizes the SHAP method to interpret model outputs by quantifying the contribution of each variable to predictions. This interpretability framework provides two dimensions of analysis: global interpretation at the feature level and local interpretation at the individual level. In the global interpretation, the SHAP summary graph (Figure 6A) illustrates the significance of each feature within the model. The results indicate that age, surgery time, anesthesia method, calcium levels, and ESR characteristics significantly influence prediction outcomes, with age exerting the most substantial impact, followed by ESR and surgery time, respectively. Notably, the violin plot (Figure 6B) reveals that elevated values of age, surgery time, and ESR generally exert a positive influence, whereas lower values tend to negative effect. Additionally, patients undergoing general anesthesia contribute positively to the predictions. In contrast, high calcium levels are linked to negative contributions, while low calcium levels yield positive contributions. Meanwhile, the decision plot (Figure 6C) and heat map (Supplementary Figure 3) also illustrated the impact of each indicator on the model output.

**Figure 7:**
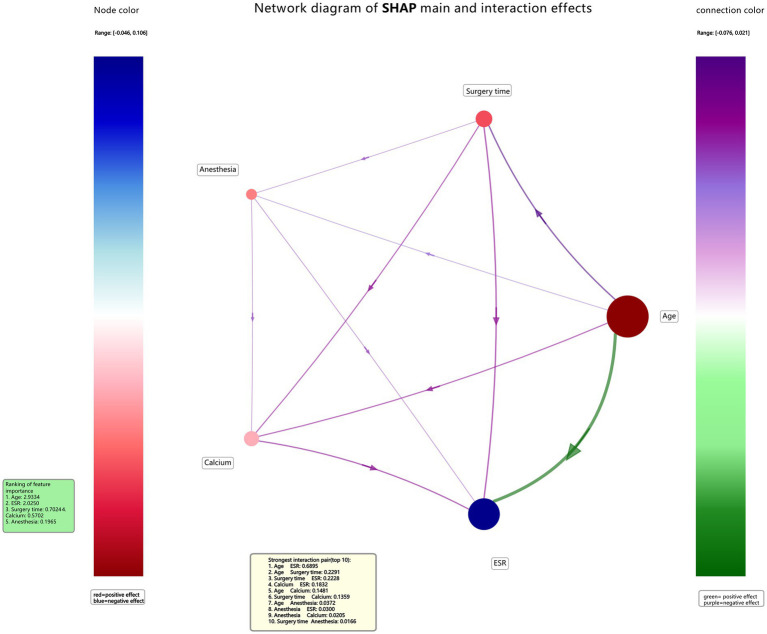
Dependence plot showing the relationship the age feature and its SHAP value, with adjuvant as a color-coded interaction feature. The *x*-axis represents the age feature value, and the *y*-axis represents its SHAP value.

SHAP dependence plots demonstrate the nonlinear relationships between each feature and model output (Supplementary Figure 4). Age and ESR were positively associated with SHAP values, indicating that higher values of these variables increased model prediction outputs. Surgery time exhibited a U-shaped relationship with SHAP values, while Calcium showed a nonlinear fluctuating trend. Additionally, interaction effects were observed among variables, especially between Age and ESR, suggesting that their combined influence contributed to the final prediction of the model (Figure 7; Supplementary Figures 5–9).

**Figure 8:**
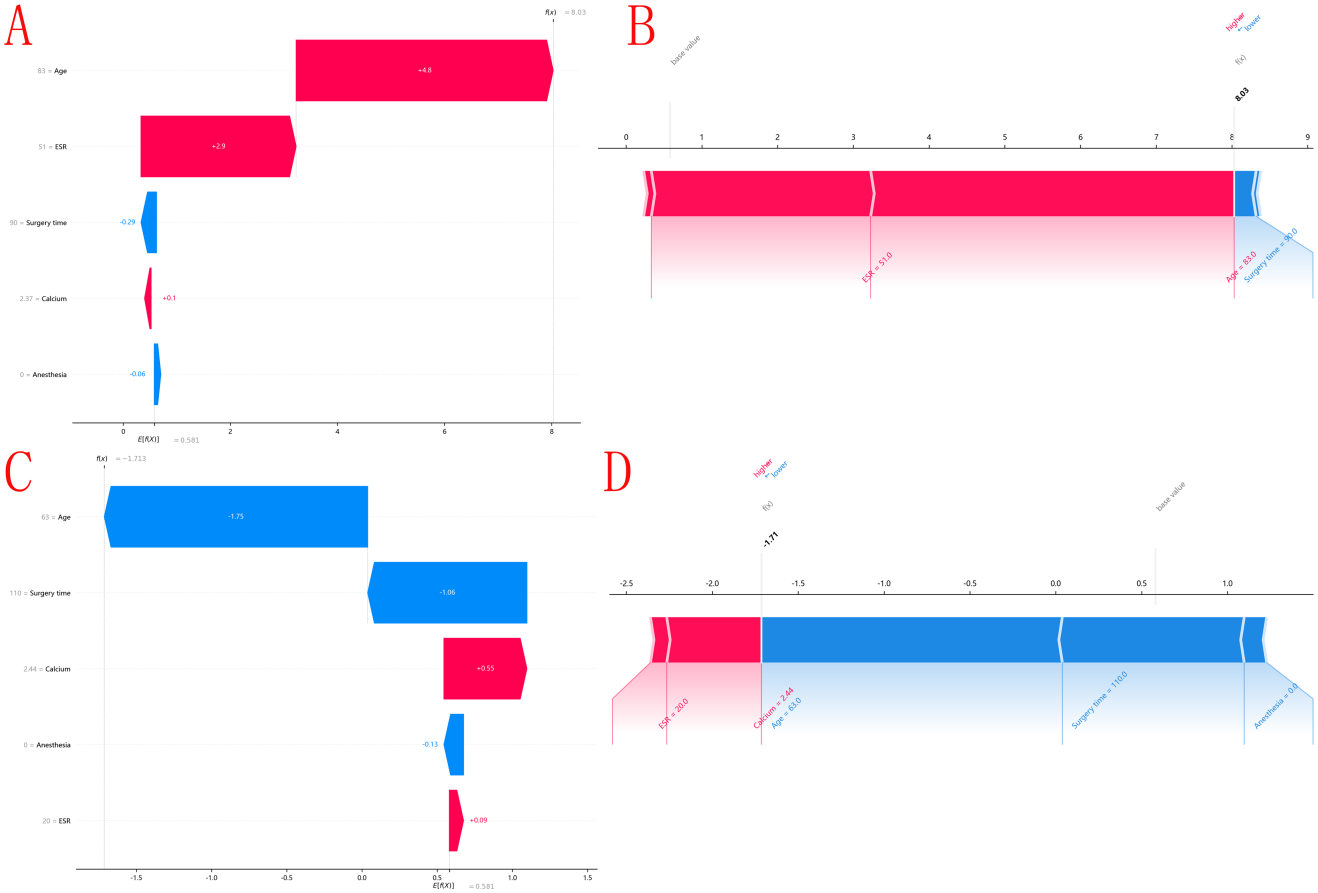
Local model explanation by the SHAP method. **(A,B)** Waterfall and force plots showing the evolution of risks contributed by each feature for individuals with TJA at low risk of postoperative hypoproteinemia. **(C,D)** Waterfall and force plots showing the evolution of risks contributed by each feature for individuals with TJA at high risk of postoperative hypoproteinemia. The base value (E[*f*(*x*)]) represents the average mode output, while the arrows indicate how each feature contributes to pushing the prediction [*f*(*x*)] toward either the postoperative hypoproteinemia or not.

The SHAP waterfall and force plots offer a detailed, individualized explanation of the decision-making process of the ML model, illustrating how specific clinical characteristics influence personalized risk assessments. In the case of the high-risk patient (final prediction *f*(*x*) = 8.03, the corresponding probability is 99.97%), as shown in Figures 8A,B, the model’s output was significantly elevated above the baseline expectation (E [*f*(*x*)] = 0.581) primarily due to two major positive factors: advanced age (83 years, contributing +4.8 to the prediction) and elevated erythrocyte sedimentation rate (ESR = 51, contributing +2.9). Conversely, factors such as surgery duration, calcium levels, and type of anesthesia had minimal counteractive effects, with surgery duration and anesthesia slightly decreasing the predicted risk, while calcium had a marginal increasing effect. This underscores the significance of age and systemic inflammation (as indicated by ESR) as the key determinants of high-risk categorization in this particular scenario. However, for the low-risk patient (final prediction [*f(x)*] = −1.713, the corresponding probability is 15.28%) (Figures 8C,D), the model’s output was significantly reduced below the baseline (E [*f(x)*] = 0.581) primarily due to a strong negative influence from age (63 years, −1.75). This protective effect was further enhanced by a negative impact from surgery time (110 min, −1.06) and a minor adverse effect from anesthesia type (−0.13). Conversely, calcium level (+0.55) and ESR (+0.09) had positive effects, aiming to elevate the predicted risk. Nevertheless, these positive influences were insufficient to counterbalance the potent negative factors, resulting in an overall low-risk forecast. This illustrates that, despite slightly elevated calcium and ESR levels, a combination of younger age and a less-than-expected impact of surgery time can decisively lead to a low predicted risk. These findings highlight the model’s capability to capture diverse, patient-specific risk profiles, emphasizing that while age and ESR consistently play crucial roles in predictions, their effects depend on the context and their interactions with other clinical variables, such as surgery time, can significantly influence the final risk evaluation. This level of interpretability is crucial for translating model outcomes into actionable clinical insights.

### Accessible web application for clinical utility

To improve clinical applicability, we have created an interactive web application utilizing the Streamlit framework, which converts the optimized prediction model into a practical tool. Clinicians can simply enter the actual values of the five key features of the model to receive immediate individualized risk predictions for postoperative hypoproteinemia. The network forecasting tool is accessible at https://hypoproteinemia-prediction-uzhpp3jadq8ryey4jxq3qe.streamlit.app/. It is important to note that this model was developed and validated using data from two hospitals in Southwest China; therefore, users are encouraged to validate its performance in their own patient populations before routine clinical use.

## Discussion

This study developed and validated a machine learning predictive model that integrates clinical and laboratory data to assess the risk of hypoproteinemia in middle-aged and elderly patients following TJA. In contrast to previous researches that primarily utilized LR model or focused solely on conventional clinical variables, this investigation systematically explores and compares the performance of eight ML algorithms for this prediction task. These algorithms include LR, KNN, DT, RF, XGBoost, LightGBM, SVM, and CatBoost. By analyzing multi-dimensional characteristic variables, the study identified 5 key predictive factors: age, surgery time, ESR, anesthesia method, and serum calcium level. Notably, the LightGBM model exhibited the highest *AF_score_* (0.858) and demonstrated superior performance across standard evaluation metrics, achieving AUC values of 1.000, 0.968, and 0.855 in the training, internal validation, and external validation cohorts, respectively. The near-perfect AUC of 1.000 in the training cohort suggests potential over-fitting, which is a known limitation of complex machine learning models. However, the acceptable performance in the external validation cohort (AUC = 0.855) supports the model’s generalizability. This finding suggests that as an ensemble learning technique, LightGBM can effectively manage high-dimensional data while maintaining promising predictive capabilities. To enhance the interpretability and applicability of the model in clinical practice, this study innovatively employed the SHAP method to provide a transparent interpretation of the ML model and to visualize the contribution of each variable to the risk of postoperative hypoproteinemia. The analysis revealed that age, surgery time, and ESR are positively correlated with the risk of postoperative hypoproteinemia. Additionally, the risk significantly increases in patients undergoing general anesthesia, while the preoperative serum calcium level exhibits a negative correlation with this risk. Utilizing visualization tools such as waterfall plots, the study presented the prediction results for two random cases, with probabilities of postoperative hypoproteinemia at 15.28 and 99.97%, respectively. This visualization clarified the specific impact of clinical variables on individual risk and effectively addressed the prevalent “black box” issue associated with ML models.

This study identified age as the most significant risk factor for predicting hypoproteinemia following joint replacement surgery. The results of the SHAP analysis (Figure 6) demonstrate that age contributes most substantially to the model’s predictive outcomes. This finding aligns with established clinical understanding and the conclusions of numerous prior studies (21, 31–33). From a pathophysiological perspective, the heightened risk of postoperative hypoproteinemia in elderly patients (≥60 years old) can be attributed to two primary factors. On the one hand, aging is associated with physiological changes that result in a general decline in digestive and absorptive functions. The atrophy of the gastrointestinal mucosa reduces the efficiency of protein intake and absorption. Consequently, the preoperative basal albumin reserve is often already low, complicating the body’s ability to manage protein consumption during postoperative stress (34, 35); On the other hand, the synthetic function of the liver diminishes in elderly patients, and the half-life of albumin is extended. Furthermore, after TJA surgery, the body experiences high metabolic stress due to inflammatory responses from surgical trauma and an increased demand for tissue repair. This situation exacerbates the “imbalance between supply and demand” for albumin synthesis in the liver, ultimately resulting in decreased serum albumin levels (36). In addition, elderly patients frequently present with multiple chronic underlying conditions, such as heart and kidney insufficiency and diabetes, which can disrupt protein metabolic homeostasis. When these conditions are compounded by surgical interventions, they produce a cumulative effect that significantly elevates the risk of hypoproteinemia (37, 38). Notably, the SHAP dependence analysis (Supplementary Figures 4, 5) indicates that the relationship between age and the risk of postoperative hypoproteinemia is not merely linear; it encompasses complex nonlinear dynamics. Specifically, the risk escalates rapidly before the age of 60, reaches a plateau between ages 60 and 70, and then exhibits a diminishing marginal risk contribution after age 70. This nuanced finding offers greater clinical insight than the simplistic assertion that “risk increases with age.” It may illustrate the “healthy survivor effect” or selection bias in clinical practice; that is, ultra-elderly patients who can endure surgery and live beyond 80 years are likely a relatively healthy cohort that has undergone natural selection and rigorous preoperative evaluation, resulting in superior physiological reserves and compensatory capabilities compared to their average peers. Simultaneously, this implies that clinicians may have subconsciously implemented cautious perioperative approaches for extremely elderly patients, including enhanced nutritional support, meticulous intraoperative procedures, and rigorous postoperative surveillance, potentially mitigating age-related risks. This finding underscores the importance of not solely relying on age as a determinant in risk evaluation for elderly patients, but rather advocating for a holistic assessment considering their overall physiological state and the quality of perioperative care provided.

This study identified that an elevated preoperative ESR serves as an independent risk factor for hypoproteinemia following TJA, with its significance ranking just below that of age (Figure 6). As a well-established inflammatory marker, an elevated ESR level directly indicates the presence of a systemic or localized inflammatory state. Furthermore, prior research has demonstrated that inflammation constitutes a critical contributor to hypoproteinemia (39). A clear pathophysiological link exists between the mechanisms of inflammation and the onset of postoperative hypoproteinemia (40): surgical trauma activates the innate immune system, triggers a systemic inflammatory response, and releases pro-inflammatory cytokines such as interleukin-6 (IL-6) and tumor necrosis factor-*α* (TNF-α). These inflammatory mediators influence albumin metabolism through two primary pathways: first, they directly inhibit the expression and translation of albumin mRNA in liver cells, thereby reducing its synthesis rate; second, they increase the permeability of capillary endothelial cells, facilitating the leakage of significant amounts of albumin from blood vessels into the interstitial spaces, which results in a marked decrease in serum albumin concentration. Consequently, patients with elevated ESR levels prior to surgery often indicate a “pre-stimulated” inflammatory baseline. In this context, the additional stress imposed by the surgical procedure is likely to trigger an amplification effect characterized by an “inflammatory storm,” significantly heightening the risk of postoperative hypoproteinemia (41).

The relationship between decreased serum calcium levels (typically <2.2 mmol/L) and hypoproteinemia following TJA has been inadequately explored in prior research, although the findings of this model indicate its potential clinical relevance. From a metabolic perspective, calcium serves as an “essential coenzyme factor” in protein synthesis within the liver, facilitating the activation of amino acids and the elongation of peptide chains during albumin synthesis. A decline in serum calcium levels inhibits the function of liver ribosomes, which directly reduces the rate of albumin synthesis. Furthermore, patients undergoing TJA often experience decreased appetite and inadequate calcium intake due to pain-related stress. Concurrently, the stress response elicited by surgical trauma leads to increased calcium excretion by the kidneys, thereby exacerbating the hypocalcemic state and creating a detrimental cycle of “hypocalcemia-hypoprotein” (42, 43). Consequently, it is imperative in clinical practice to routinely monitor serum calcium levels prior to surgery, provide calcium supplementation for patients with low levels, and dynamically maintain calcium balance postoperatively to foster an optimal metabolic environment for albumin synthesis.

Prolonged operation time is a well-established risk factor for postoperative hypoproteinemia. This phenomenon can be attributed to the continuous traumatic stress and anesthesia exposure associated with longer surgeries, which lead to increased secretion of stress hormones, such as cortisol, and heightened protein catabolism. Concurrently, intraoperative bleeding and fluid loss are exacerbated. When crystalloid fluids are predominantly used for supplementation, they can result in blood dilution, thereby directly reducing serum albumin concentration (44). Furthermore, extended operation times are frequently linked to more complex surgical procedures, which themselves induce greater trauma and elevate the risk of postoperative infections. This creates a chain reaction characterized by “prolonged operation time—aggravated trauma/infection—increased protein consumption.” Additionally, the choice of anesthesia influences postoperative risks. Compared to regional anesthesia, general anesthesia may elicit more pronounced systemic stress responses and the release of inflammatory factors, potentially affecting early recovery of gastrointestinal function and nutrient intake post-surgery, thus adversely impacting both protein consumption and supply. Hence, it is crucial for clinicians to efficiently manage surgical duration through optimizing procedures and improving technical skills, especially for complex surgical procedures. Key strategies involve meticulous preoperative planning to minimize unnecessary tissue damage, judiciously combining colloid and crystalloid fluids during surgery for effective volume control to mitigate blood dilution’s direct impact on serum albumin levels. Furthermore, selecting and optimizing anesthesia plans, such as prioritizing regional block anesthesia, within the confines of surgical safety requirements, can significantly reduce patients’ metabolic risks during the perioperative phase.

Meanwhile, we identified significant interaction effects among ESR levels, operation time, calcium ion concentrations, anesthesia methods, and age factors. In elderly patients, elevated ESR, prolonged operation time, reduced calcium ion levels, or alterations in anesthesia methods can substantially increase the risk of hypoproteinemia associated with aging. For example, an elderly patient with a high preoperative ESR faces a markedly greater postoperative risk than what would be anticipated from advanced age or elevated ESR alone. This finding underscores the efficacy of machine learning models in elucidating complex nonlinear relationships. It indicates to clinicians that, when evaluating the risks for elderly patients, a comprehensive assessment of inflammatory markers (such as ESR), operation time, calcium ion levels, and anesthesia methods in conjunction with age is essential. Patients exhibiting abnormal indicators, particularly elderly individuals, should be classified as extremely high-risk groups for postoperative hypoproteinemia. In addition to routine monitoring, appropriate preventive measures or clinical interventions should be considered to enhance patient outcomes.

The advantages of this research are primarily evident in several key areas. First, it introduces an innovative methodology. Unlike traditional statistical approaches, this study employs a system of multiple ML algorithms to predict the risk of hypoproteinemia following TJA. It effectively utilizes the SHAP framework to elucidate the complex nonlinear relationships and interaction effects among various predictive factors. SHAP analysis addresses the prevalent “black box” limitations of ML models by intuitively quantifying the contribution of each feature to individual prediction outcomes. This significantly enhances the clinical interpretability and credibility of the model, offering a novel perspective for a deeper understanding of the multifactorial mechanisms underlying this complication. Second, the model achieves a commendable balance between high performance and practical applicability. The constructed lightGBM model exhibits robust and stable discriminative performance in both internal and external validations (AUC 0.968 and 0.855). Furthermore, to enhance clinical applicability, we developed an interactive web application using the Streamlit framework, thereby transforming the optimized predictive model into a practical tool. Clinicians can input the actual values of the 5 key features of this model to obtain immediate individualized risk predictions for postoperative hypoproteinemia after TJA. Third, the research design is notably rigorous. Through multi-center data collection, stringent patient inclusion and exclusion criteria, and the division of data into training sets, internal validation sets, and external validation sets, the reliability of the research findings and the generalizability of the model have been significantly enhanced.

The observed associations between the five predictors (age, ESR, surgery time, anesthesia method, and serum calcium) and postoperative hypoproteinemia should be interpreted as predictive factors rather than causal determinants. While our SHAP analysis revealed nonlinear relationships and interaction effects among variables, these findings are hypothesis-generating and require confirmation in prospective etiological studies. The model’s predictive performance, although promising, does not imply clinical superiority over existing risk assessment approaches without direct comparative trials.

### Limitations

This study has several limitations. Firstly, the retrospective research design is susceptible to selection bias and information bias. Despite efforts to control for these biases, there may be unmeasured confounding factors in the medical records (such as subtle differences in nutritional status and unrecorded inflammatory states), preventing confirmation of causal relationships. The observed associations should be interpreted as predictive factors rather than causal determinants. Secondly, the near-perfect AUC of 1.000 in the training cohort suggests potential over-fitting, a known risk with complex machine learning models. Although external validation (AUC = 0.855) supports generalizability, prospective validation in larger, independent cohorts is warranted. Thirdly, the generalizability of the model requires further validation. The mean age (65–69 years) and sex distribution of the 1,383 participants were broadly consistent with previously reported TJA populations in China. However, both participating hospitals are located in Southwest China, and patients were predominantly of Han ethnicity. Therefore, the findings may not be directly generalizable to other ethnic groups, healthcare systems, or populations with different nutritional or comorbidity profiles. Fourthly, several potentially relevant predictors identified in the literature were not available for analysis due to the retrospective design and limitations of the electronic medical records. These include preoperative serum albumin level (which was an exclusion criterion), detailed nutritional assessment tools (e.g., Mini Nutritional Assessment), frailty indices, socioeconomic status, and postoperative laboratory trends. Future prospective studies incorporating these variables may further improve the predictive performance of the model. Fifthly, the *AF_score_* evaluation metric proposed in this study is exploratory and has not been formally validated. Independent methodological studies are needed to establish its robustness before widespread adoption. Nevertheless, in this study, it served as an important integrated measure alongside established metrics (AUROC, calibration curves, and DCA) for comprehensive model evaluation. Sixthly, although the model demonstrated acceptable performance in an external validation cohort from a second hospital, this validation was performed on a single independent cohort from the same geographic region (Southwest China). The calibration curve of the external validation data was suboptimal, likely due to the limited sample size. Widespread clinical implementation of the model is not recommended before further validation in diverse, multi-center, and international populations. Readers are encouraged to assess the model’s performance in their own patient settings. Seventhly, the study solely examined short-term postoperative outcomes, neglecting the model’s predictive ability for long-term postoperative results. Lastly, enhancements are needed in the clinical integration and dynamic prediction capabilities of the model. Presently, the model heavily relies on static preoperative and immediate intraoperative indicators, failing to integrate laboratory parameters and evolving clinical events postoperatively. Future research could explore incorporating continuous monitoring data to develop a dynamic risk prediction model encompassing the entire perioperative period.

## Conclusion

This study successfully developed and validated a machine learning prediction model characterized by high accuracy and interpretability. This model not only predicts the risk of hypoproteinemia following TJA in middle-aged and elderly patients but also elucidates the complex nonlinear relationships among various factors. It may serve as a potential tool for the early prediction and screening of patients at elevated risk for postoperative hypoproteinemia, with the potential to shift postoperative management strategies from passive correction to active prevention based on early risk assessment. However, prospective, multi-center studies are needed to further validate its clinical utility before widespread implementation.

## Data Availability

The original contributions presented in the study are included in the article/Supplementary material, further inquiries can be directed to the corresponding authors.
