# Supplementary material for: Interpretable machine learning model for predicting postoperative hypoproteinemia in middle-aged and elderly patients following total joint arthroplasty: a multicenter retrospective study
Source: Front Nutr. 2026 Jun 12;13:1775504. doi: 10.3389/fnut.2026.1775504 (PMC13303208; doi:10.3389/fnut.2026.1775504)
Supplement: Supplementary file 1 [file Data_Sheet_1.ZIP › Supplementary materials.doc]

**Supplementary materials**

**Supplementary Table 11.** Results of Univariate analysis for external validation cohorts.

| **Characteristics** | **External Test Cohort** | | | |
| --- | --- | --- | --- | --- |
|  | **Overall**  **N =548** | No  (N=235) | Yes  (N=313) | **P-value** |
| **Gender, n(%)** |  |  |  | <0.001 |
| Male | 186 (33.9%) | 52 (22.1%) | 134 (42.8%) |  |
| Female | 362 (66.1%) | 183 (77.9%) | 179 (57.2%) |  |
| **Age (years), Mean ± SD** | 69 ± 10 | 60 ± 6 | 76 ± 6 | <0.001 |
| **BMI (Kg/m^2^), Mean ± SD** | 24.9 ± 7.4 | 25.6 ± 10.5 | 24.3 ± 3.6 | 0.067 |
| **Hypertension, n(%)** |  |  |  | <0.001 |
| Yes | 233 (42.5%) | 75 (31.9%) | 158 (50.5%) |  |
| No | 315 (57.5%) | 160 (68.1%) | 155 (49.5%) |  |
| **Diabetes, n (%)** |  |  |  | 0.040 |
| Yes | 88 (16.1%) | 29 (12.3%) | 59 (18.8%) |  |
| No | 460 (83.9%) | 206 (87.7%) | 254 (81.2%) |  |
| **Cardiovascular disease, n (%)** |  |  |  | <0.001 |
| Yes | 65 (11.9%) | 14 (6.0%) | 51 (16.3%) |  |
| No | 483 (88.1%) | 221 (94.0%) | 262 (83.7%) |  |
| **Hyperlipidaemia, n (%)** |  |  |  | 0.963 |
| Yes | 37 (6.8%) | 16 (6.8%) | 21 (6.7%) |  |
| No | 511 (93.2%) | 219 (93.2%) | 292 (93.3%) |  |
| **Hyperuricemia, n (%)** |  |  |  | 0.632 |
| Yes | 72 (13.1%) | 29 (12.3%) | 43 (13.7%) |  |
| No | 476 (86.9%) | 206 (87.7%) | 270 (86.3%) |  |
| **ASA, n (%)** |  |  |  | <0.001 |
| ≥III | 269 (49.1%) | 57 (24.3%) | 212 (67.7%) |  |
| <III | 279 (50.9%) | 178 (75.7%) | 101 (32.3%) |  |
| **General anesthesia, n (%)** |  |  |  | 0.109 |
| Yes | 31 (5.7%) | 9 (3.8%) | 22 (7.0%) |  |
| No | 517 (94.3%) | 226 (96.2%) | 291 (93.0%) |  |
| **Surgical method, n (%)** |  |  |  | 0.793 |
| THA | 330 (60.2%) | 143 (60.9%) | 187 (59.7%) |  |
| TKA | 218 (39.8%) | 92 (39.1%) | 126 (40.3%) |  |
| **Surgery time (minutes), Mean ± SD** | 93 ± 28 | 97 ± 32 | 91 ± 25 | 0.029 |
| **RBC, Mean ± SD** | 4.21 ± 0.56 | 4.34 ± 0.54 | 4.11 ± 0.56 | <0.001 |
| **HGB, Mean ± SD** | 126 ± 16 | 131 ± 16 | 123 ± 16 | <0.001 |
| **MCV, Mean ± SD** | 92 ± 7 | 93 ± 7 | 92 ± 7 | 0.317 |
| **MCH, Mean ± SD** | 30.19 ± 2.90 | 30.29 ± 2.78 | 30.11 ± 2.98 | 0.459 |
| **MCHC, Mean ± SD** | 327 ± 12 | 327 ± 12 | 327 ± 13 | 0.780 |
| **NLR, Mean ± SD** | 4.12 ± 4.45 | 3.06 ± 3.23 | 4.92 ± 5.04 | <0.001 |
| **PLR, Mean ± SD** | 164 ± 91 | 149 ± 69 | 176 ± 104 | <0.001 |
| **LMR, Mean ± SD** | 3.74 ± 1.88 | 4.29 ± 1.89 | 3.33 ± 1.76 | <0.001 |
| **SII, Mean ± SD** | 837 ± 909 | 655 ± 744 | 973 ± 994 | <0.001 |
| **SIRI, Mean ± SD** | 2.05 ± 2.89 | 1.40 ± 2.21 | 2.54 ± 3.22 | <0.001 |
| **PIV, Mean ± SD** | 422 ± 613 | 313 ± 542 | 503 ± 650 | <0.001 |
| **ALI, Mean ± SD** | 409 ± 324 | 507 ± 390 | 335 ± 240 | <0.001 |
| **PT, Mean ± SD** | 11.22 ± 1.40 | 11.07 ± 1.77 | 11.32 ± 1.02 | 0.055 |
| **INR, Mean ± SD** | 0.97 ± 0.13 | 0.96 ± 0.18 | 0.98 ± 0.07 | 0.187 |
| **APTT, Mean ± SD** | 26.4 ± 4.1 | 26.3 ± 4.5 | 26.5 ± 3.8 | 0.707 |
| **TT, Mean ± SD** | 17.71 ± 3.02 | 17.86 ± 1.75 | 17.59 ± 3.68 | 0.258 |
| **FIB, Mean ± SD** | 3.27 ± 0.98 | 3.14 ± 0.97 | 3.37 ± 0.97 | 0.008 |
| **AST/ALT, Mean ± SD** | 1.25 ± 0.53 | 1.13 ± 0.53 | 1.34 ± 0.51 | <0.001 |
| **Calcium, Mean ± SD** | 2.25 ± 0.13 | 2.37 ± 0.07 | 2.16 ± 0.09 | <0.001 |
| **CRP, Mean ± SD** | 13 ± 25 | 9 ± 19 | 16 ± 29 | <0.001 |
| **ESR, Mean ± SD** | 34 ± 26 | 30 ± 25 | 37 ± 27 | <0.001 |
| **PNI, Mean ± SD** | 47.7 ± 4.6 | 49.3 ± 4.3 | 46.5 ± 4.3 | <0.001 |
| **HS-mGPS, n (%)** |  |  |  | <0.001 |
| 0 | 265 (48.4%) | 135 (57.4%) | 130 (41.5%) |  |
| 1 | 283 (51.6%) | 100 (42.6%) | 183 (58.5%) |  |
| **NAR, Mean ± SD** | 0.12 ± 0.06 | 0.10 ± 0.06 | 0.13 ± 0.07 | <0.001 |
| **CAR, Mean ± SD** | 0.34 ± 0.66 | 0.22 ± 0.49 | 0.42 ± 0.76 | <0.001 |

Note: BMI, body mass index; THA, total hip arthroplasty; TKA, total knee arthroplasty; ASA, American Society of Anesthesiologists score; RBC, red blood cell; HGB, hemoglobin; MCV, mean corpuscular volume; MCH, mean corpuscular hemoglobin; MCHC, mean corpuscular hemoglobin concentration; NLR, neutrophil to lymphocyte ratio; PLR, platelet to lymphocyte ratio; LMR, lymphocyte to monocyte ratio; SII, systemic immune-inflammatory index; SIRI, systemic inflammatory response index; PIV, pan-immune-inflammation value; ALI, advanced lung cancer inflammation index; PT, prothrombin time; APTT, activated partial thrombin time; INR, international normalized ratio; TT, thrombin time; FIB, fibrinogen; CRP, C-reactive protein; ESR, erythrocyte sedimentation rate; PNI, Prognostic Nutritional Index; HS-mGPS, High-sensitive Modified Glasgow Prognostic Score; NAR, neutrophil to albumin ratio; CAR, CRP to albumin ratio;

**Supplementary Table 2*2*:** The coefficients of Lasso regression analysis in training cohort.

| variable | Coefficient |
| --- | --- |
| (Intercept) | -8.703983129 |
| Gender | 0.000000000 |
| Age | 0.129987771 |
| BMI | 0.000000000 |
| Hypertension | 0.000000000 |
| Diabetes | 0.000000000 |
| Cardiovascular.disease | 0.000000000 |
| Hyperlipidaemia | 0.000000000 |
| Hyperuricemia | 0.000000000 |
| ASA | 0.000000000 |
| Surgical.method | 0.000000000 |
| Surgery.time | 0.006996059 |
| Anesthesia | -0.597593288 |
| RBC | 0.000000000 |
| HGB | 0.000000000 |
| MCV | 0.000000000 |
| MCH | 0.000000000 |
| MCHC | 0.000000000 |
| NLR | 0.000000000 |
| PLR | 0.000000000 |
| LMR | 0.000000000 |
| SII | 0.000000000 |
| SIRI | 0.000000000 |
| PIV | 0.000000000 |
| ALI | 0.000000000 |
| PT | 0.000000000 |
| INR | 0.000000000 |
| APTT | 0.000000000 |
| TT | 0.000000000 |
| FIB | 0.000000000 |
| AST/ALT | 0.000000000 |
| Calcium | -0.245036151 |
| CRP | 0.000000000 |
| ESR | 0.046554683 |
| PNI | 0.000000000 |
| HS.mGPS | 0.000000000 |
| NAR | 0.000000000 |
| CAR | 0.000000000 |

Note: BMI, body mass index; ASA, American Society of Anesthesiologists score; RBC, red blood cell; HGB, hemoglobin; MCV, mean corpuscular volume; MCH, mean corpuscular hemoglobin; MCHC, mean corpuscular hemoglobin concentration; NLR, neutrophil to lymphocyte ratio; PLR, platelet to lymphocyte ratio; LMR, lymphocyte to monocyte ratio; SII, systemic immune-inflammatory index; SIRI, systemic inflammatory response index; PIV, pan-immune-inflammation value; ALI, advanced lung cancer inflammation index; PT, prothrombin time; APTT, activated partial thrombin time; INR, international normalized ratio; TT, thrombin time; FIB, fibrinogen; CRP, C-reactive protein; ESR, erythrocyte sedimentation rate; PNI, Prognostic Nutritional Index; HS-mGPS, High-sensitive Modified Glasgow Prognostic Score; NAR, neutrophil to albumin ratio; CAR, CRP to albumin ratio;

**Supplementary Table 3. TRIPOD Checklist for Prediction Model Studies.**

| **Section/Topic** | **Item** | **Checklist Item** | **Page** |
| --- | --- | --- | --- |
| ****Title & Abstract**** | 1 | Title | 1 |
|  | 2 | Provide summary of objectives, design, setting, participants, sample size, predictors, outcome, analysis, results | 2-3 |
| ****Introduction**** | 3a | Explain medical context and rationale | 3-5 |
|  | 3b | Specify study objectives | 5-6 |
| ****Methods**** | 4a | Describe study design and data source | 6 |
|  | 4b | Specify key study dates | 6 |
|  | 5a | Describe study setting (number of hospitals, region) | 6 |
|  | 5b | List eligibility criteria | 6-7 |
|  | 5c | Treatments before outcome assessment | N/A |
|  | 6a | Define outcome (albumin <35 g/L) | 6 |
|  | 6b | Outcome assessment and blinding | 6 |
|  | 7a | Define all predictors | 7-8 |
|  | 7b | Predictor assessment and blinding | N/A |
|  | 8 | Sample size justification (EPV rule) | 6 |
|  | 9 | Missing data handling (complete case analysis) | 6 |
|  | 10a | Predictor selection (literature + LASSO) | 8-9 |
|  | 10b | Predictor handling in modeling | 9 |
|  | 10c | Model development (8 ML algorithms, 5-fold CV) | 9-11 |
|  | 10d | Performance measures (AUROC, AUPRC, calibration, DCA, AF_score_) | 10-11 |
|  | 10e | Model updating | N/A |
|  | 10f | Hyper-parameter tuning | 10 |
|  | 10g | Code availability | 26 |
|  | 11 | Risk group classification | N/A |
|  | 12 | Differences between development and validation | 6 |
| ****Results**** | 13a | Participant flow | 12 |
|  | 13b | Participant characteristics | 12, Table 1 |
|  | 13c | Comparison of development and validation cohorts | Table 1 |
|  | 14 | Number with missing data | 6 |
|  | 15 | Predictor selection results | 13, Figure 2 |
|  | 16 | Model performance per cohort | 13-14, Figures 3-5 |
|  | 17 | Model updating results | N/A |
| ****Discussion**** | 18 | Performance vs. previous studies | 17-22 |
|  | 19a | Study advantages | 22-23 |
|  | 19b | Overall interpretation | 22 |
|  | 19c | Study limitations | 23-25 |
|  | 20 | Conclusion | 25 |
| ****Other**** | 21 | Funding sources | 26 |

**Supplementary Figure 1.** PR curve plot of 8 different Machine Learning models (A: training cohort; B: internal validation cohort; C: external validation cohort).

**Supplementary Figure 2.** Radar plot of 8 different Machine Learning models (A: training cohort; B: internal validation cohort; C: external validation cohort).

**Supplementary Figure 3.** Heat plot of LightGBM model.

**Figure 4.** Dependence plot of 8 different Machine Learning models in (A: age; B: ESR; C: surgery time; D: calcium).

**Supplementary Fig 5.** Interaction plot showing the relationship the age feature and its SHAP value, with adjuvant as a color-coded interaction feature. The x-axis represents the age feature value, and the y-axis represents its SHAP value.

**Supplementary Fig 6.** Interaction plot showing the relationship the ESR feature and its SHAP value, with adjuvant as a color-coded interaction feature. The x-axis represents the ESR feature value, and the y-axis represents its SHAP value.

**Supplementary Fig 7.** Interaction plot showing the relationship the surgery time feature and its SHAP value, with adjuvant as a color-coded interaction feature. The x-axis represents the surgery time feature value, and the y-axis represents its SHAP value.

**Supplementary Fig 8.** Interaction plot showing the relationship the calcium feature and its SHAP value, with adjuvant as a color-coded interaction feature. The x-axis represents the calcium feature value, and the y-axis represents its SHAP value.

**Figure S9.** Dependence plot showing the relationship the anesthesia feature and its SHAP value, with adjuvant as a color-coded interaction feature. The x-axis represents the anesthesia feature value, and the y-axis represents its SHAP value.
